# Droplet-based Biosensing for Lab-on-a-Chip, Open Microfluidics Platforms

**DOI:** 10.3390/bios6020014

**Published:** 2016-04-14

**Authors:** Piyush Dak, Aida Ebrahimi, Vikhram Swaminathan, Carlos Duarte-Guevara, Rashid Bashir, Muhammad A. Alam

**Affiliations:** 1 Purdue University, West Lafayette 47906, IN, USA; pdak@purdue.edu (P.D.); sebrahim@purdue.edu (A.E.); 2 University of Illinois at Urbana-Champaign, Urbana 61801, IL, USA; vikhram2@illinois.edu (V.S.); duarteg2@illinois.edu (C.D.-G.); rbashir@illinois.edu (R.B.)

**Keywords:** droplet, biosensors, lab-on-a-chip, early detection, point-of-care, high sensitivity

## Abstract

Low cost, portable sensors can transform health care by bringing easily available diagnostic devices to low and middle income population, particularly in developing countries. Sample preparation, analyte handling and labeling are primary cost concerns for traditional lab-based diagnostic systems. Lab-on-a-chip (LoC) platforms based on droplet-based microfluidics promise to integrate and automate these complex and expensive laboratory procedures onto a single chip; the cost will be further reduced if label-free biosensors could be integrated onto the LoC platforms. Here, we review some recent developments of label-free, droplet-based biosensors, compatible with “open” digital microfluidic systems. These low-cost droplet-based biosensors overcome some of the fundamental limitations of the classical sensors, enabling timely diagnosis. We identify the key challenges that must be addressed to make these sensors commercially viable and summarize a number of promising research directions.

## 1. Introduction

Management of many life-threatening diseases, such as cancer, tuberculosis, AIDS, malaria, and others, requires rapid, easy to use, integrated, and cheap diagnostic devices for detection of biomolecules [1,2]. The recent technological advances in microfluidics and nanotechnology present new opportunities for development of lab-on-a-chip (LoC) systems to perform a complete set of biomedical assays to achieve cost-effective, high-throughput, sensitive, point-of-care diagnostics.

**Figure 1:**
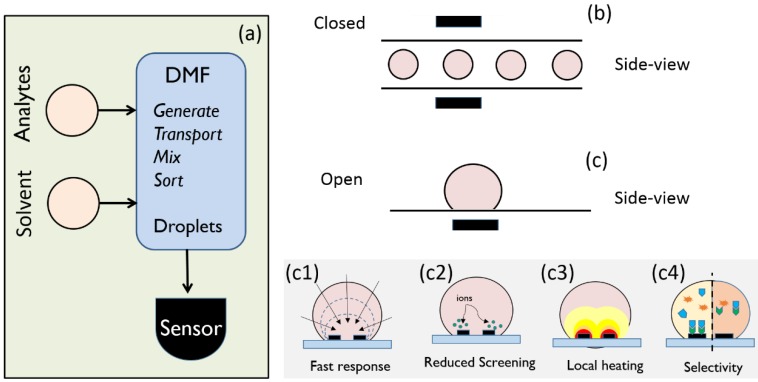
A droplet-based LoC platform must be integrated with highly sensitive and selective sensors. (**a**) General configuration of digital microfluidics platforms. Digital microfluidics offers a broad range of droplet operations (e.g., generation, transport, mixing, sensing, *etc.*). This review focusses on droplet-based sensors and their performance limits. (**b**) In a closed microfluidic system, sensors analyze the droplets as they flow past the sensors; (**c**) In an open microfluidic system, the droplet is placed on the sensor surface, and no continuous flow is required. Figure 1(**c1**–**c4**) show various aspects of droplet-based sensors covered in this article.

Over the past two decades, there have been numerous reports of microfluidic systems integrated onto a LoC platform [3,4,5,6]. Among them, digital microfluidics (DMF) offers a comprehensive set of fluidic operations, such as dispersing, transport, mixing, merging and splitting by programmable activation of a series of actuation electrodes [6,7,8], as shown in Figure 1a. DMF retains the advantages of traditional, continuous-flow microfluidic systems, namely, small sample volume, reduced reagent consumption and waste production, rapid analysis, and portability. Moreover, compared to other techniques, DMF systems operate at lower power, and are amenable to parallel processing and data acquisition for high throughput screening [8,9,10,11,12,13,14,15]. Being highly reconfigurable, DMF-based systems also satisfy the needs of various biochemical applications, e.g., chemical and enzymatic reactions, immunoassays, proteomics, DNA detection, single-cell studies, and so on [7,12,13,16,17,18,19,20,21].

The goal of any LoC technology, eventually, is to achieve fast and highly sensitive detection of a specific analyte with the smallest possible sample/reagent volume at comparatively low cost. However, these otherwise sophisticated LoC technologies often rely on relatively simple sensors, e.g., colorimetric, rudimentary flow cytometry, UV-Vis absorbance spectroscopy, *etc.* [22,23,24]. In several applications, the technology, as is, has had enormous impact; for others, more sensitive sensors, that are also compatible with the architecture and topology of droplet microfluidics, are desired [5,25]. For example, real-time, rapid detection of sub-femtomolar concentration of biomolecules is critical in various areas, such as, biomedical diagnostics/therapeutics, food safety, environmental monitoring, and homeland security. The traditional sensors that achieve such high degree of sensitivity are usually too large for integration with microfluidic systems, moreover, the process technologies are often incompatible. Therefore, the recent development of droplet-based biosensors compatible with the architecture of a DMF platform has attracted considerable attention in biomedical research and applications, especially in drug screening, biomarker analysis, and on-chip chemical synthesis [5,8].

There are two types of droplet-based sensing platforms. For closed-microfluidic systems (Figure 1b), the sensors straddle the channel, collecting data as droplets flow past the sensor. Such systems are well developed and offer high throughput and simple integration. In contrast, open microfluidics shown in Figure 1c involves planar (often, multifunctional) sensors where analytes within the droplets are interrogated. Typically, an open microfluidic system is simpler and cheaper to fabricate, easier to reconfigure, and offers faster sample handling and direct access to droplets for analyte extraction, if necessary [6].

Given the novelty of the open-microfluidic droplet-based biosensors, it is important to assess their performance in terms of the three fundamental metrics of biosensors: response time, sensitivity, and selectivity [26]. In this review, our goal is to summarize the efforts of various groups to improve these metrics (shown graphically in Figure 1(c1–c4)) and address the challenges of droplet-based sensors. For other components of the microfluidic systems (e.g., manipulation and washing/purification which are schematically shown in Figure 1a), we refer the readers to several excellent reviews on the topic [8,27,28,29]. Finally, label-free sensors are desired for all bio-assays, so that the analyte molecule need not be first be attached to a “label-molecule” for subsequent detection. Label-free approaches reduce time and cost of sample preparation significantly. In this review, therefore, we focus on label-free, droplet-based biosensors.

### 1.1. Response Time of Biosensors

Response time ($t_{s}$) is defined as the minimum time needed for a biosensor to capture sufficient amount of biomolecules to identify an analyte. Regardless of the detection mechanism, $t_{s}$ is limited by the physical diffusion of molecules towards the sensor surface [3,5,18]. In practice, $t_{s}$ can be extraordinarily long at low analyte concentrations (ρ): Even the most sensitive nanowire (NW)-biosensor would need more than a day to positively identify an analyte at 1 fM concentration [30].

Fortunately, there are several ways to reduce the response time. Recall that the response time reduces for higher analyte concentration, ρ≜N/V, where N is the number of analyte molecules and V is the sample volume. In one approach, ρ is increased by increasing N, through Polymerase Chain Reaction (PCR) or Circular Strand-Replacement Polymerization (CSRP) [31,32,33]. The N-amplification approach is adopted by several commercial assays, e.g., Ion Torrent (Thermo Fisher Scientific, Waltham, MA, USA) [31]. These approaches are very sensitive and selective, but are expensive, need long preprocessing time, and require trained personnel and complex instrumentation which is likely to limit their applicability in fast, point-of-care (PoC) diagnosis [34]. The second approach is to increase ρ is by reducing V, e.g., as in biobarcode assay [35] and droplet evaporation on open DMF [9,11]. We will see in Section 2 that droplet evaporation offers a simple, yet efficient way to significantly reduce $t_{s}$ and improve sensitivity, even for ultra-low analyte concentrations (see Figure 1(c1)).

### 1.2. Screening-Limited Sensitivity of Biosensors

Potentiometric biosensors, which detect the analyte charge directly, allow label-free detection and are easily miniaturized [36,37,38,39,40]. Since the target molecules conjugate with the probe molecules (usually immobilized on the sensor surface as shown in Figure 1(c4), left) only in salt-based electrolyte solutions, screening by these ions fundamentally limits the sensitivity of charge-based (potentiometric) biosensors. The length-scale over which the charges are screened is given by the Debye length, $\lambda_{D}=\sqrt{\varepsilon k_{B}T/2i_{0}q^{2}}$, where ε is the dielectric permittivity, $k_{B}$ the Boltzmann constant, T the temperature, q the fundamental electronic charge, and $i_{0}$ the ionic strength of the electrolyte. Ionic strength of physiological fluids, such as blood and plasma, is in the range of 135 mM−140 mM, for which $\lambda_{D}<1\;nm$. Since a sensor cannot effectively “see” the biomolecules located at a few Debye lengths away, its sensitivity to those biomolecules is dramatically reduced [41].

Various approaches have been adopted to mitigate this fundamental screening-limited sensitivity of potentiometric sensors. Commonly used techniques include detection in low-ionic strength electrolytes, either by performing binding-sensing steps at low ionic strength [42] or using a flow-through apparatus that performs the binding and the sensing steps at different ionic strengths [43]. Both the approaches, however, reduce the binding affinity of the target molecule to the immobilized probe, which may degrade selectivity (the ability of a sensor to differentiate between target *vs.* parasitic molecules). Other approaches include detection of biomolecular dipoles by using high-frequency measurements [44] or engineering antibody capture fragments to bind the analytes close to the sensor surface [45]. Unfortunately, at present, these techniques are neither cost-effective, nor easily integrated into a droplet-based platform.

As we will see in Section 3, droplets offer a fundamentally different approach to desalting: Due to finite number of ions in a sub-nL droplet, it is possible to temporarily desalt the droplet electrically near the sensor region (graphically shown in Figure 1(c2)) to maximize the sensitivity. Swaminathan *et al.* demonstrated a method for localized electronic desalting on a field effect transistor (FET) biosensor by using on-chip polarizable electrodes to locally deplete salt ions near the sensor region [46]. Theoretical analysis by Dak *et al.* shows that such approach could lead to a 250X improvement of the detection limit [47].

### 1.3. The Importance of “Selectivity” for Integrated Biosensors

The ability to differentiate between the analyte *vs.* parasitic molecules resembles the challenge of finding a needle in a haystack. Many groups have reported highly sensitive sensor technologies, only to find that the sensor responds exquisitely to all molecules, thereby rendering the technology irrelevant and useless.

Traditionally, there are three general techniques to improve selectivity. First, and perhaps the most popular method, is the use of amperometric sensors to detect analytes. These sensors monitor the current associated with oxidation or reduction of electroactive species involved in the recognition process. Since the electroactive species is specific to the target biomolecule, amperometric sensors have a very high specificity. The second approach relies on the sample purification to capture the analytes of interest and release them in the sensing solution. For example, Stern *et al.* developed a micropurification chip that captures the cancer biomarkers (antigens) from blood and, after washing, releases the antigens into a pure buffer solution to be detected by a silicon nanoribbon sensor [48]. Similarly, Krisvitsky *et al.* used antibody-modified silicon nanowires (SNWs) to capture the target proteins, followed by subsequent release and detection using SNW-based FET arrays [49]. Finally, the third approach focuses on reducing non-specific binding by covering the gaps among receptors by small molecules, see Reference [50] for a quantitative analysis. In Section 4, we will discuss two new strategies discussed in the literature to assess the selectivity in droplet-based sensors: Localized heating (schematically shown in Figure 1(c3)) and monitoring differential binding dynamics without probe immobilization (shown in Figure 1(c4), right).

With this background on response time, sensitivity, and selectivity of classical sensors, we will now discuss in the next three sections, how the droplet-based sensors address these issues and discuss the remaining challenges before the sensors are integrated onto a droplet microfluidic platform.

## 2. Droplet-Based Beating of diffusion Limit in Electrical Biosensors

As mentioned in Section 1, droplet-based biosensors offer new approach (an alternative to number-amplification methods, such as PCR) to improve the response time by an effective increase of analyte concentration through volume reduction. As an emerging field, several research groups have used droplet evaporation to speed up biomolecules’ physical diffusion. For example, De Angelis *et al.* showed that evaporation of a microliter-sized droplet on a specially designed superhydrophobic surface (created by combination of photolithography and electron-beam lithography) locally delivers a few copies of λ–DNA to an integrated Surface-Enhanced Raman Scattering (SERS) sensor [9].

**Figure 2:**
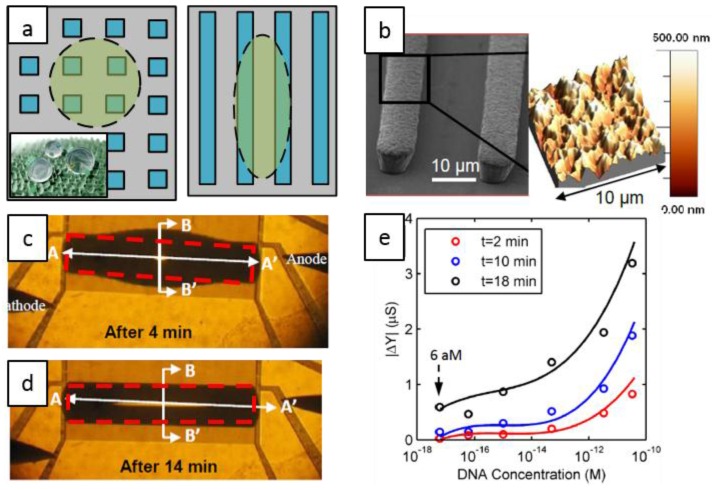
(**a**) Left: On a symmetric surface, a droplet forms a semispherical cap-shaped structure with a circular contact line. Right: It forms an oval-shaped contact line on an asymmetric surface, such as the structure in Reference [11]. The inset shows computer graphic of a lotus leaf surface; (**b**) SEM image of the electroplated electrodes. The figure on the right shows an AFM profile of the electrodes’ nanotextured surface; (**c**) An optical image of a droplet on the electrode array 4 min after deposition; (**d**) The same droplet 10 min later; (**e**) The relative conductance change as a function of the initial DNA concentration. Figures are reproduced from Reference [67] by permission of the Royal Society of Chemistry. Inset of Figure 2a is reprinted with permission from @ William Thielicke.

Similarly, there is another new class of electrical sensors that can be integrated with “open” digital microfluidics. Focusing on electrical biosensing, the authors in Reference [11] showed that time-multiplexed, droplet-based non-Faradic impedance sensing (DNFIS) succeeds in detection of attomolar-level concentration of DNA molecules [51,52]. In contrast to Faradaic EIS, no additional reagent or reference electrode are required, rendering non-Faradaic schemes somewhat more amenable to PoC applications [53,54,55,56,57,58]. The authors showed that by relying on the entire time-dependent impedance reading and intentional pinning of the droplet, the results are statistically robust, with very little uncertainly in the concentration [11]. Given its novelty, we discuss the approach in some detail below.

(a). Surface engineering to combine the “lotus effect” and the “coffee ring effect”:

The sensor relies on the ability to concentrate the biomolecules through controlled evaporation of droplets. To achieve well-defined evaporation profile, many research groups have attempted to mimic the “lotus effect” [59], by artificially creating hydrophobic surfaces with symmetric patterns, see Figure 2a (left). Unfortunately, the droplet moves around easily on such surface (as on a lotus leaf) and pinning the droplet to a location is difficult [9]. Additionally, most of the reported (super)hydrophobic surfaces are made of/coated with materials that are not electrically conductive [60,61,62]. Therefore, an electrically-conductive, hydrophobic surface which mimics a “coffee ring” (pinned edges) is required [26]. To achieve this, the authors designed asymmetric rough electrodes (that pins the droplets perpendicular to the array, but allows it to elongate parallel to it) as shown in Figure 2a (right). The fabrication process proceeds as follows. Briefly, following the deposition of the electroplating seed layer (Ni/Ti) and formation of the mold layer by standard photolithography, Ni electroplating at specific current density created the sensing array shown in Figure 2b. The hierarchical nanoscale features of the surface morphology, formed as a result of metal electroplating, are essential for pinning of the droplet so that impedance measurements are always reproducible [11]. Figure 2c,d show a droplet 4 min and 14 min after deposition, respectively, with the contact line pinned.

(b). Evaporation improves sensitivity:

Evaporation of an analyte-containing droplet (such as DNA or bacteria) changes the ionic concentration of the droplet [63,64]. As a result, the droplet conductance increases with time (Figure 2e). By continuously monitoring the impedance, one obtains several data points in a single measurement, the average of which shows very little variation [11,64,65,66]. A detection limit (DL) of 60 aM (with a response time of 18 min) was reported which is a 4–5 orders of magnitude improvement compared to bulk-based, classical non-Faradaic methods. Theory and modeling of the droplet-based non-Faradaic impedance sensing have been extensively discussed in Reference [66]*.*

Although, evaporation improves sensitivity dramatically and the sensitivity is maximized in solutions with low conductivity (to increase the impedance contrast), some biomolecules (e.g., proteins, double stranded DNA, red blood cells, *etc.*) are very sensitive to the changes of conductivity and retain their properties only in specific ionic conditions. Fortunately, the theory predicts and the experiments confirm that non-Faradic EIS shows excellent sensitivity even at high salt concentration at suitably high measurement frequency. Another approach involves time-dependent modulation of salt concentration to be discussed in the following section.

## 3. Droplets to Overcome Screening Limit

We explained in Section 1 that charge screening by salt ions limits the potential of charge-based sensors. The finiteness of droplets in a droplet-based sensor suggests new opportunities to combat screening. Specifically, due to finite number of ions in a droplet, it is possible to temporarily desalt the droplet electrically near the sensor region to transduce larger fraction of biomolecule charge to the sensor. Theoretical work in Reference [47] shows that ~50 pL droplets can be appreciably desalted for physiological concentrations using high-surface area electrodes. Droplet desalting for such a system, has been experimentally demonstrated in Reference [46] for concentrations ≤10 mM.

**Figure 3:**
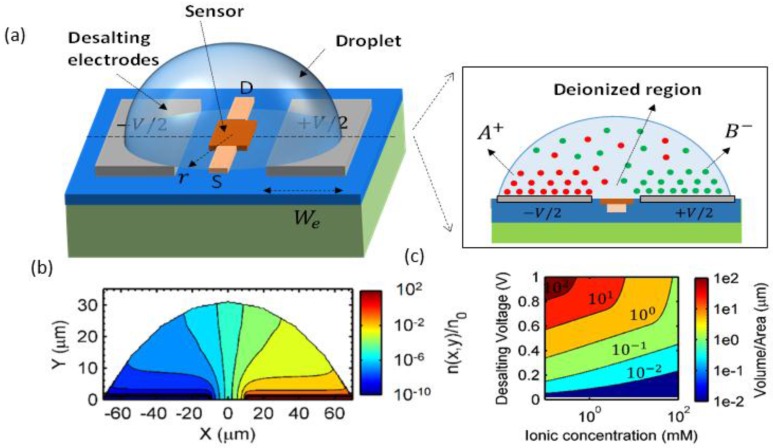
(**a**) Schematic of a FET/nanowire biosensor with on-chip electrodes for localized desalting and simultaneous device biasing. Positive $\left(A^{+}\right)\;$and negative $\left(B^{-}\right)$ ions are attracted towards negative and positive polarity electrodes, respectively, depleting the droplet bulk of salt; (**b**) Numerical calculation of ion profile showing negative ion density in a 300 pL droplet (6100 $\mu m^{2}$ electrode area) at 1 μM (background strength under 1 V desalting bias); (**c**) Ratio of the droplet- volume to the electrode-area required for desalting the droplet by 50%, as a function of desalting voltage and ionic concentration. For example, desalting at 100 mM concentration under 1 V desalting bias requires an aspect ratio of ~1 μm . Reproduced with permission from *Appl. Phys. Lett*. 106, 053105 (2015). Copyright 2015, AIP Publishing LLC.

Figure 3a shows the approach used by the authors to desalt sub-nL droplets placed on a set of polarizable coplanar electrodes surrounding the sensing unit. The electrodes were fabricated by conventional evaporation and lift-off patterning of 1000 Å thick Ti/Pt films. A DC bias (less than the over-potential) is applied across the electrodes to adsorb the excess ions within the electrical double layer (EDL). A transistor at the center of the droplet can be used for chemical/biomolecule sensing. Due to small volume of the sample, the droplet is desalted without undesirable parasitic effects, such as redox reactions, gas bubbling, and/or heating. Figure 3b shows the numerical simulation of the negative ion density profile within a 300 pL droplet for 1 μM initial concentration at an applied bias of 1 V. The ions pile up near the electrodes and, consequently, deplete the droplet bulk to less than 1% of the original ionic concentrations.

The authors generalized the analysis by Kilic *et al.* [68] to droplet-based systems in order to determine the extent of desalting in a droplet. The desalting efficiency is related to the droplet volume (V), ionic strength$\;(i_{o}$), the bias across the desalting electrodes ($V_{e}$), and the electrode surface area (A). Figure 3c shows the ratio of droplet volume to electrode area (V/A) that is required for desalting droplets of various salt concentrations to a fraction of *f* = 0.5; clearly, V/A ratio varies considerably with the ionic concentration to be desalted. To desalt more feasible and addressable droplets (≥100 *pL*) at concentrations up to 10 mM, the authors used nanostructured Pt-black to increase A. Briefly, electrodeposition of the Pt-black electrode was done on a seed layer of 1000 Å thick Ti/Pt from dihydrogen hexachloroplatinate at a specific current density to obtain highly branched dendritic nanostructures. They reported 50% desalting for droplets with salt concentration of 10.8 mM. Theoretical calculations in Reference [47] show that with 100X enhancement of electrode area, the detection limit can be improved by almost 250X. However, the ability to engineer electrodes with such a larger effective area remains an important research problem.

In addition to addressing the screening issue, droplet desalting can also be used to modulate the DNA denaturation (unzipping) temperature [47]. This could open up opportunities to conduct PCR at room temperature, with modulation of salt concentration as a proxy for temperature control. The technical feasibility of such an approach to either reduce screening in sensors and/or modulate the DNA denaturation temperature define interesting future research direction for the field.

## 4. Selectivity in Droplet-Based Systems: DNA Hybridization as a Case-Study

The third important consideration for droplet-based biosensors is their ability to positively identify an analyte, in the presence of parasitic molecules. Detection of specific DNA molecules through hybridization of target DNA to probe molecules is an important component of many bioassays, such as detection of cancer, bacterial infection, viral infections, *etc.* Therefore, in this section we focus on droplet-based assays that targeted DNA hybridization.

A number of techniques have been proposed, such as, optical tagging, surface plasmon resonance (SPR), mechanical resonance sensors, field-effect transistors, and electrochemical impedance spectroscopy [69,70,71,72,73,74,75,76,77,78,79,80,81]. Section 1.3 summarized several traditional approaches to selectively detect a particular analyte. Briefly, to address selectivity, most biosensors contain a bio-recognition layer (e.g., aptamers, antibodies, T-phages, *etc.* [82,83,84,85]) which is immobilized on the sensor surface or the surface of nano/micro-particles, as shown in Figure 1(c4) (left). In part (a), section, we discuss the use of traditional *immobilization-based* scheme for detection of DNA hybridization (using fluorescence spectroscopy as the transduction method) in a droplet-based system. In part (b), we discuss the use of *immobilization-free* scheme for detection of DNA hybridization (based on non-Faradaic impedance sensing as the transduction mechanism) in droplet-based system.

(a) Localized heating for performing biochemical reactions necessary for selective detection:

In this section, we discuss a scheme to detect single-base pair mismatch in DNA by changing its binding state through an on-chip heating mechanism. This scheme, as discussed earlier, relies on traditional immobilization-based method.

In order to perform on-chip heating, several approaches have been used, such as Peltier heaters, resistive heaters, microwave heaters, *etc.* [86,87,88,89,90]. These method are either not amenable to small droplet sizes, or do not allow heating of individual droplets. In addition, they usually require oil encapsulation to suppress evaporation, which limits on-chip integration.

In an effort to address these issues, Salm *et al.* presented an on-chip miniaturized FET-based dielectric heating scheme to control the temperature within the droplet locally [67]. This method allowed parallel heating of sub-nanoliter droplets and did not require any encapsulation layer for minimizing evaporation. The authors used on-chip heating in conjunction with Fluorescence resonant energy transfer (FRET) scheme to detect single-base mismatch between DNA in picoliter-sized droplets.

The technique relies on modifying the DNA strand and its complementary strand with fluorescein (FAM) and a black hole quencher (BHQ), respectively. In the double stranded conformation, there is transfer of energy between FAM and BHQ and hence the observed fluorescence is smaller as compared to single stranded conformation. The binding state of a probe-target pair is changed by heating the sample solution to different temperatures using the on-chip heater, and fluorescence is measured as a function of the control variable which determines the temperature. This temperature (or the control variable) is used as a proxy for determining whether the probe–target pair are complementary or not.

**Figure 4:**
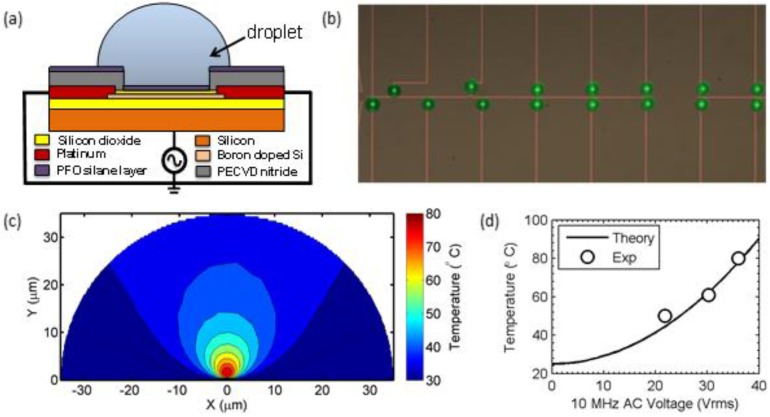
(**a**) Schematic of a droplet sitting on top of a FET device; (**b**) An array of droplets sitting on linked devices for parallel detection; (**c**) Simulated temperature profile within the droplet for an applied bias 36 V. Temperature within the droplet is highly localized, and returns close to room temperature at the edges minimizing the evaporation; (**d**) Theoretical estimate of the droplet temperature as a function of applied ac bias. Temperature varies roughly as a square of the applied AC bias. (Figure 4b–c are adapted and Figure 4d replotted from Reference [67] with permission from National Academy of Sciences).

Figure 4a shows the schematic of the dielectric heating device used for heating the droplet. For fabricating the device, authors used a top-down procedure starting with a silicon-on-insulator (SOI) wafer. To reduce the active layer thickness, part of the layer was oxidized and etched using buffered oxide etchant. This was followed by lithography and reactive ion etching to define the active areas. After source/drain doping, silicon oxide was grown to form the gate oxide and metal contacts were defined by lift-off. Finally, a nitride-rich plasma enhanced chemical vapor deposition layer was deposited and pattered to expose device channel and probing pads. Detailed fabrication steps can be found in Reference [67].

An AC voltage is applied between the transistor’s leads and the bulk substrate. Figure 4b shows an array of droplets sitting on linked devices for parallel detection. The authors provide a self-consistent numerical model with electrical and thermal equations to determine the spatial and temporal temperature profile within the droplet. Figure 4c shows the numerical simulation of the thermal profile within a 30 μm radius droplet. Due to the localized nature of fringing fields around the device, the heating of the droplet is highly localized and occurs at the core of the droplet. The temperature at the perimeter of the droplet returns close to the room temperature, which, fortunately, minimize evaporation. The authors show that the temperature within the droplet varies as the square of the applied bias (see Figure 4d), and the temperature is stabilized within milliseconds of the onset of the AC voltage.

**Figure 5:**
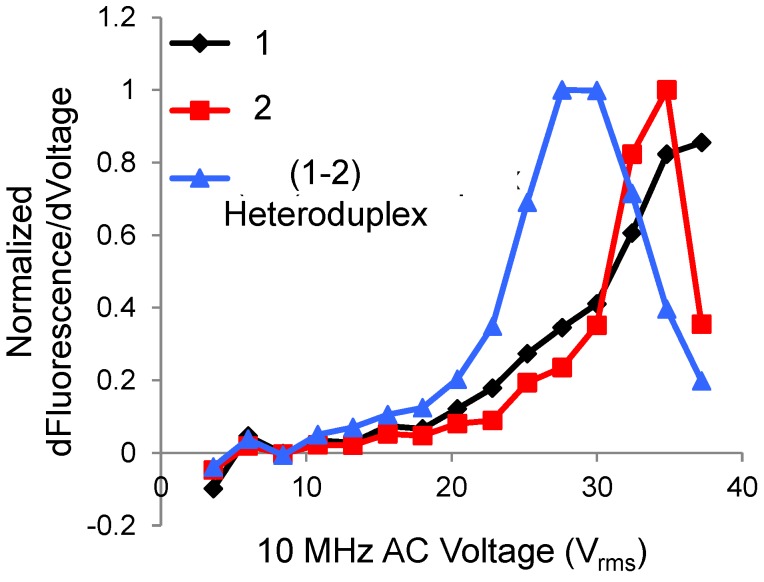
Derivative of fluorescence w.r.t. voltage *vs.* AC voltage for 3 DNA strands, the red and black curves correspond to DNA samples with fully-complementary strands and the blue curve a hetroduplex with a single-base pair mismatch. The hetroduplex showed the peak at lower voltage, thereby indicating a single-base pair mismatch (because of lower melting temperature). Figure replotted from Reference [67] with permission from National Academy of Sciences.

Using the approach, the authors first perform a parallel nucleic acid denaturation study, and then use fluorescence based detection method to determine the single-base mismatch. Figure 5 shows the melting curve analysis performed on a set of three different DNA strands. DNA strands with a single-base mismatch have lower overall free energy leading to a reduced melting temperature (equivalently, less applied bias) as compared to the one without mismatch.

While the immobilization-based technique described in this sub-section offers good selectivity, surface functionalization requires several hours of incubation and use of specific chemicals, and is also known to reduce the hybridization efficiency by a factor of 20–40 [80]. Further, since the sensor depends on end-point detection, it suffers from the diffusion limited sensor response. In order to overcome these limitations, an immobilization-free scheme relying on droplet-evaporation may be used, which is discussed next.

(b) DNFIS as a DNA hybridization assay

As a label-free, electrical scheme, Reference [65] reports application of DNFIS (discussed in Section 2) as a selective, immobilization-free DNA hybridization sensor. The authors showed that 22-mer *unamplified* specific DNA strands can be distinguished at concentration of 2 nM, in a little more than an hour.

The basic idea is as follows: the conductance of DNA-containing solutions changes when two ssDNA molecules conjugate to form a dsDNA strand [91,92]. As reported by many groups [64,91,92,93,94], the higher the density of ssDNA, the higher the conductance of the solution. This is presumably because the condensed ions are released to the loosely surrounding ion cloud in transition from dsDNA to ssDNA. A partially matched dsDNA, therefore, is characterized by a conductance in between ssDNA and fully-matched dsDNA.

**Figure 6:**
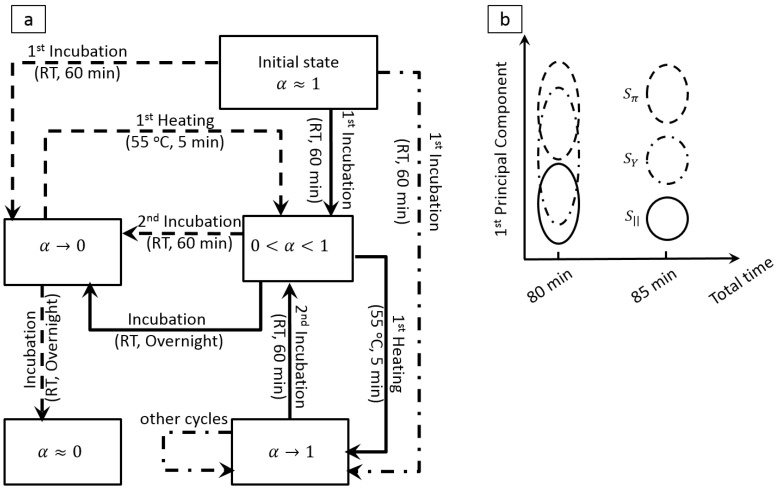
(**a**) State-machine shows how various solution evolve as their binding state changes through time. Solid, dashed, and dotted-dashed lines represent $S_{\pi }$ (full match), $S_{Y}$ (partly-match), and $S_{\left|\right|}$ (full-mismatch) solutions, respectively; (**b**) Plot of the first principal component obtained from (i) a data set comprised of the results of the initial state and 1st incubation (total evaluation time of 80 min), and (ii) by considering the results obtained from the 1st heating step to the data set (total time ~85 min). Selective detection down to 2 nM is realized after ~85 min.

To determine the binding state of solutions with different levels of base-pair mismatch, the solutions go through a series of carefully chosen incubation/heating steps to repeatedly break and reform the conjugates, and capture the modulation in the binding state through modulation of the droplets′ impedance. Depending on the degree of base-pair mismatch and the kinetics of the transition from ssDNA to dsDNA (and vice versa), the solutions undergo different transition paths, as shown in Figure 6a. The temperature cycling modulates the ratio of the ssDNA molecules to the total DNA density (α) and thereby the total measured impedance. Since modulation of α depends on the degree of mismatch between the strands, modulation of the measured impedance identifies the target DNA and its concentration.

The impedance values at each step create a dataset with at least 5 variables (from each cycle) for each solution. The authors analyzed the high-dimensional data by principal component analysis (PCA) and demonstrated that adding only one heating step (additional 5 min) is sufficient for selective detection of the target DNA strand (Figure 6b). In addition, they showed that by using PCA, the linear operation range of the sensor improves by two orders of magnitude [65].

To summarize, in this section we have discussed two techniques for selective detection of DNA molecules in droplets. Both techniques rely on determining the conformational state of probe-target pair to selectively detect DNA molecules. The on-chip heating methodology uses an on-chip heater to change the conformational state and then selectively determines the DNA using FRET based detection. In contrast, the droplet-based impedance sensing method uses repeated off-chip heating cycles to change the conformational state and selectively detect the molecules using an on-chip impedance based detection. One future research direction could be to integrate these two schemes onto a fully functional LoC platform.

## 5. Challenges and Outlook

Despite significant advances discussed above, it is fair to say that selectivity remains a key concern for biosensors in general, and droplet-based biosensors in particular. Two approaches could improve the state of the art considerably.

(a). Pre-filtration by functionalization:

With recent advances in open digital microfluidics, various components of a high throughput biosensing assay (e.g., sample preparation, manipulation, transport, heating, amplification, and detection) can be performed in parallel on a single chip. In addition, in order to get the full advantages of DNFIS (e.g., time-multiplexed data acquisition, elimination of reference electrode, ultrahigh sensitivity, *etc.*) one could functionalize microbeads or nanoparticles with the biorecognition material, followed by their release in a separate spot on the open platform (similar to biobarcode assay [35]). The target analytes can then be transported to the detection spot, which can be DNFIS.

(b). Development of miniaturized, on-chip reference electrodes:

Among various label-free approaches, Faradaic impedance and field-effect transistor (FET) based sensors offer excellent selectivity. However, as mentioned earlier, a reference electrode is necessary to stabilize the fluid potential in both of these methods. Conventional reference electrodes are bulky, fragile and too big to be inserted into a droplet. Therefore, in order to extend the capability of droplet-based impedance sensing and to enable charge-based detection in desalting systems, a miniaturized reference electrode must be integrated. While an ideal miniaturized reference electrode has not been developed, several research groups [95,96,97] have demonstrated miniaturized quasi-reference electrodes which could potentially be integrated into LoC platforms.

## 6. Conclusions

To summarize, one of the major roadblocks to commercialization of droplet-based screening systems is the ability to combine different steps, such as, sample collection, sample treatment, analyte-specific reaction, signal generation and detection on a single platform. Component design and fabrication procedures must evolve to ensure that different modules are compatible with each other, and are able to function together. The paper discussed the emergence of droplet-based biosensors as a promising technology to overcome some of the fundamental limitations of the bulk-based sensing systems, such as diffusion limit, response time, and screening. The rapid advances in digital microfluidics for massively parallel handling, manipulation, analyte amplification, and analysis of millions of droplets further pave the way for realization of high throughput, label-free electrical screening of biological entities for applications such as fast drug screening, personal proteomics, *etc.* Being compatible with architecture of the “open” DMF systems, once the challenges associated with selectivity of electrical droplet-based biosensors are addressed properly, their integration will dramatically broaden the application space of the LoC technologies for highly sensitive, on-demand, low-cost screening.
